# POLYCYSTIC OVARY SYNDROME: ORIGINS AND IMPLICATIONS: Genetics of polycystic ovary syndrome (PCOS)

**DOI:** 10.1530/REP-25-0126

**Published:** 2025-09-26

**Authors:** Yvonne V Louwers, Jenny A Visser, Andrea Dunaif, Joop S E Laven

**Affiliations:** ^1^ Division of Reproductive Endocrinology and Infertility, Department of Obstetrics and Gynecology, Erasmus University Medical Center, Rotterdam, The Netherlands; ^2^ Department of Internal Medicine, Erasmus MC, University Medical Center Rotterdam, Rotterdam, The Netherlands; ^3^ Division of Endocrinology, Diabetes and Bone Disease, Icahn School of Medicine at Mount Sinai, New York, New York, USA

**Keywords:** PCOS, GWAS, Mendelian randomization, cluster analysis, next-generation sequencing

## Abstract

Polycystic ovary syndrome (PCOS) is a common and heterogeneous disorder currently diagnosed only in reproductive-age women. Familial clustering and twin studies have provided strong evidence for a genetic contribution to PCOS pathogenesis. First-degree relatives, including males and non-reproductive-age females, have reproductive and metabolic phenotypes consistent with a genetic susceptibility to these traits. PCOS is now recognized as a complex trait influenced by both genetic and environmental factors. Genome-wide association studies have identified ∼30 loci linked to PCOS, implicating pathways involved in gonadotropin secretion and action, folliculogenesis, steroidogenesis, age at menopause, and carbohydrate metabolism. Next-generation sequencing has found rare variants in *AMH*, *AMHR2*, and *DENND1A*, supporting these genes’ central role in developing PCOS. Epigenetic mechanisms, such as DNA methylation and non-coding RNAs, influence gene regulation and may contribute to phenotypic heterogeneity. Unsupervised clustering has identified distinct reproductive and metabolic subtypes with unique genetic architectures, providing a biologically meaningful framework for classification. This shift from expert opinion-based diagnosis to data-driven classification has the potential to transform PCOS management and enable precision medicine approaches tailored to distinct subtypes of the disorder.

## Introduction

Polycystic ovary syndrome (PCOS) is a highly heritable, heterogeneous disorder affecting 5–20% of reproductive-age women worldwide, depending on the diagnostic criteria applied (Knochenhauer *et al.* 1998, Diamanti-Kandarakis *et al.* 1999, Broekmans *et al.* 2006, Carmina & Azziz 2006, Goodarzi & Azziz 2006, Chen *et al.* 2008, Yildiz *et al.* 2012, Lauritsen *et al.* 2014). It is the leading cause of anovulatory infertility. PCOS is commonly associated with insulin resistance and obesity, disorders that confer increased risk for type 2 diabetes (T2D) (Rubin *et al.* 2017) as well as for other serious cardiometabolic morbidities across the lifespan (Diamanti-Kandarakis & Dunaif 2012). Nevertheless, the cause(s) of PCOS remain unknown, and the disorder is relatively understudied compared to other common medical conditions affecting women (Brakta *et al.* 2017). PCOS is now recognized as a complex genetic trait in which phenotype results from susceptibility genes interacting with environmental factors (Visscher *et al.* 2017, Visscher *et al.* 2021, Dapas & Dunaif 2022). Genetic analyses have substantially advanced our understanding of PCOS’s biologic underpinnings. Indeed, more than 30 PCOS genome-wide association study (GWAS) loci have been reproducibly mapped in Han Chinese and European ancestry PCOS populations (Day *et al.* 2018, Dapas & Dunaif 2022, Tyrmi *et al.* 2022, van der Ham *et al.* 2024). PCOS-associated variants map to loci encompassing genes regulating gonadotropin secretion (Hayes *et al.* 2015) and action (Chen *et al.* 2011, Shi *et al.* 2012), androgen biosynthesis (Chen *et al.* 2011, Shi *et al.* 2012, Day *et al.* 2018), glucose homeostasis (Day *et al.* 2015, Day *et al.* 2018), folliculogenesis, and menopausal age (Day *et al.* 2015, van der Ham *et al.* 2024).

### Evidence for genetic susceptibility

Familial clustering of polycystic ovary syndrome (PCOS) was first reported in the 1960s, which suggested a genetic predisposition to the syndrome (Cooper *et al.* 1968, Judd *et al.* 1973, Cohen *et al.* 1975, Ferriman & Purdie 1979, Givens 1988). The phenotypic overlap between PCOS and rare monogenic insulin resistance syndromes prompted the investigation of insulin receptor mutations (Kahn *et al.* 1976, Stuart *et al.* 1986, Taylor *et al.* 1992, Sorbara *et al.* 1994). Familial studies confirmed the clustering of PCOS reproductive features, indicative of heritability (Lunde *et al.* 1989, Carey *et al.* 1993, Legro *et al.* 1998, Govind *et al.* 1999, Kashar-Miller & Azziz 1999, Franks *et al.* 2008). When premature balding was used to identify affected men, it suggested a dominant inheritance pattern. However, small sample sizes limited these studies (Azziz & Kashar-Miller 2000, Carey *et al.* 1993, Govind *et al.* 1999). Elevated androgen levels were prevalent in sisters, affecting approximately 40% (Legro *et al.* 1998, Govind *et al.* 1999, Franks *et al.* 2008). Bimodal distributions of non-sex hormone-binding globulin (SHBG)-bound testosterone levels in sisters suggested potential monogenic contributions or threshold-dependent feedback loops (Weinshilboum & Raymond 1977, Legro *et al.* 1998, Legro *et al.* 2002*a*, Dapas & Dunaif 2022). Brothers, mothers, and premenarchal daughters exhibited elevated androgen levels, indicating a consistent reproductive endophenotype (Legro *et al.* 2002*b*, Sam *et al.* 2006, Torchen *et al.* 2014). Elevated AMH levels were observed in male and female relatives, including children (Recabarren *et al.* 2008, Sir-Petermann *et al.* 2006, Torchen *et al.* 2016, Torchen *et al.* 2019). Metabolic features, including hyperinsulinemia, T2D, and obesity, were present in both male and female relatives (Legro *et al.* 1998, Legro *et al.* 2002*a*, Sir-Petermann *et al.* 2002, Yildiz *et al.* 2003, Sam *et al.* 2006, Coviello *et al.* 2009).

Heritability refers to the proportion of trait variation in a population that can be attributed to genetic differences among individuals (Visscher *et al.* 2008). The higher the heritability of a trait or disease, the more informative genetic studies can be in identifying its underlying causes. Twin studies are particularly valuable for estimating heritability because twins share the same environmental influences, allowing researchers to isolate genetic contributions (Conley *et al.* 2013). By comparing trait correlations between monozygotic (MZ) twins, who share identical DNA, and dizygotic (DZ) twins, who share half of their genetic material, researchers can assess the relative genetic contribution to a condition such as PCOS.

Early twin studies suggested a genetic influence on PCOS. An Australian study of 19 MZ and 15 DZ twin pairs found that while PCOS characteristics were not always identical, concordance was higher in MZ twins, indicating a genetic component (Jahanfar *et al.* 1997). The most comprehensive study, conducted using data from the Netherlands Twin Register, analyzed 1,332 MZ twins, 1,873 DZ twins, and their singleton sisters. PCOS was defined using the 2003 Rotterdam criteria, which include menstrual irregularities and clinical signs of hyperandrogenism. The study reported a concordance rate of 0.71 in MZ twins, nearly double the 0.38 observed in DZ twins, leading to an estimated additive heritability of 0.66. When accounting for individual genetic contributions from oligomenorrhea, acne, and hirsutism, the overall heritability estimate increased to 0.79 (Vink *et al.* 2006). These findings provide strong evidence for a major genetic contribution to PCOS pathogenesis.

### Genetic analyses before genome-wide association studies

Early genetic studies of PCOS primarily relied on a candidate gene approach, wherein polymorphic sites near selected genes – chosen based on their presumed involvement in disease-related pathways – were examined for associations with PCOS. However, this strategy was inherently constrained by assumptions regarding gene function. Given the key clinical features of PCOS, initial candidate genes included those implicated in steroidogenesis (e.g. *CYP11A1, CYP17A1, STAR*), androgen and gonadotropin signaling (e.g. *FSHR, LHCGR, SHBG, AR*), and insulin resistance (e.g. *INSR, INS-VNTR, IGF1, IGF1R, IRS1, PPARG*), among others (Urbanek *et al.* 1999). Although numerous associations were reported in these studies, the results were often inconsistent, with only a few findings replicated across independent studies, likely due to methodological limitations (Goodarzi *et al.* 2008, Dunaif 2016). Many studies failed to account for population stratification, leading to spurious associations driven by differences in allele frequencies across ethnic groups (Hirschhorn & Daly 2005, Bjonnes *et al.* 2016). In addition, lack of control for comorbid phenotypes, such as obesity, may have contributed to misleading association signals (Dunaif 2016). These challenges were compounded by small sample sizes, reducing statistical power to detect variants with modest effects (Kosova & Urbanek 2013). Moreover, inadequate correction for multiple hypothesis testing increased the likelihood of false-positive associations (Pau *et al.* 2013). Differences in diagnostic criteria further complicated candidate gene studies, as variations in phenotype definitions could affect the populations analyzed and the reproducibility of findings (Bjonnes *et al.* 2016, Wolf *et al.* 2018).

To evaluate the reliability of findings from PCOS candidate gene studies, Hiam *et al.* conducted a systematic review of candidate gene meta-analyses (Hiam *et al.* 2019). Among 21 meta-analyses examined, only five were classified as high quality. Many failed to clearly define inclusion criteria for control groups or exclude studies reporting statistically implausible allele distributions (Schaid & Jacobsen 1999). Furthermore, the quality assessment framework applied (Shea *et al.* 2017) did not specifically address concerns unique to genetic association studies, meaning that even the higher-quality meta-analyses remained subject to the same underlying issues affecting candidate gene research. A recent meta-analysis did identify SNPs in IL-6 and the FTO gene as risk alleles. Interestingly, a SNP in the CAPN10 gene seems to be protective for PCOS, whereas another SNP in the same gene seems to confer an increased risk for PCOS. Similarly, a SNP in the RAB5B gene was also associated with an increased risk for developing PCOS (Sharma *et al.* 2023*a*, Sharma *et al.* 2024).

The first successfully replicated genetic risk locus for PCOS was discovered in a region of chromosome 19 near the insulin receptor gene (INSR). Analyses utilizing linkage and transmission disequilibrium tests on 150 families with affected individuals identified a variant near INSR that was significantly associated with PCOS or hyperandrogenemia, based on NIH criteria (Urbanek *et al.* 1999). The decision to focus on NIH PCOS or hyperandrogenemia phenotypes stemmed from earlier research demonstrating that hyperandrogenemia represented a key endophenotype among sisters of affected women (Legro *et al.* 1998). This association was later reproduced in independent family-based cohorts (Urbanek *et al.* 2005, 2007, Stewart *et al.* 2006, Ewens *et al.* 2010) and confirmed through case–control studies (Tucci *et al.* 2001, Dapas & Dunaif 2022). While INSR was initially considered the likely causal gene, the implicated variant was located within an intron of the fibrillin-3 gene (*FBN3*), approximately 800 kb away from *INSR*. As a result, the risk allele may have been marking a variant within *FBN3* or another adjacent gene. Nevertheless, Goodarzi *et al.* later identified an intronic *INSR* variant that showed significant association with PCOS in a large case–control study involving 799 cases and 3,758 controls. This further supports INSR as the primary candidate gene within this region (Goodarzi *et al.* 2011). Additional confirmation came from GWAS (Shi *et al.* 2012, van der Ham *et al.* 2024). However, in the absence of functional validation of specific INSR variants, the precise identity of the causal gene(s) within this locus remains unresolved.

### Modern genetic analyses

#### GWAS

GWAS have proven effective in mapping common variants (minor allele frequency (MAF) ≥5%) associated with complex traits, offering critical insights into disease mechanisms (Visscher *et al.* 2017, Ingelsson & McCarthy 2018, Visscher *et al.* 2021). Although the identified variants generally have modest effect sizes and explain only a small proportion of PCOS heritability, increasing sample sizes and comprehensive analyses of all variants are expected to resolve much of the so-called ‘missing heritability’ observed in complex traits (Yang *et al.* 2010, Zaitlen & Kraft 2012).

The first GWAS was published in 2011 by a Chinese group and identified three genome-wide significant loci in three large cohorts of Han Chinese women with and without PCOS. Two loci were on the short arm of chromosome 2, and the identified single nucleotide polymorphisms (SNPs) were located near the *LHCGR* gene and a gene called *THADA*. The third one was located on the long arm of chromosome 9 and was close to a gene called *DENND1A* (Chen *et al.* 2011). A second GWAS from the same group in a larger cohort of Han Chinese women identified eight new risk loci for PCOS. These PCOS association signals showed evidence of enrichment for candidate genes related to insulin signaling, sex hormone function, T2D, calcium signaling, and endocytosis (Shi *et al.* 2012). In the same year, these authors identified another susceptibility locus encoding a gene called *YAP1*. The latter SNPs were also associated with decreased glucose tolerance and increased serum luteinizing hormone (LH) levels in women with PCOS (Li *et al.* 2012).

Two subsequent GWAS were performed in women of European ancestry, with the first using the NIH definition for PCOS (Hayes *et al.* 2015) and the second using self-reported PCOS cases validated by replication in additional cohorts of Rotterdam and NIH phenotype cases (Day *et al.* 2015). These European GWAS identified five novel associations – including a locus encompassing the FSHB gene, which encodes the beta subunit of follicle-stimulating hormone (FSH) – in addition to replicating two Han Chinese association signals. A more recent, large-scale meta-analysis of PCOS in European ancestry identified three novel loci and replicated 11 previously reported loci (Day *et al.* 2018). A cross-ethnic GWAS confirmed that 12 out of 17 genetic variants identified in the Chinese GWAS had a similar effect size and identical direction in PCOS patients from Northern European ancestry, indicating a common genetic risk profile for PCOS across populations (Louwers *et al.* 2013). A GWAS in Northern European ancestry cases replicated at least four PCOS susceptibility loci identified in the Chinese GWAS and discovered eight novel signals (Brower *et al.* 2015). Two GWAS have also been performed in Korean women using the Rotterdam criteria (Hwang *et al.* 2012, Lee *et al.* 2015), but neither identified any loci significantly associated with PCOS, likely due to limited sample sizes and the prevalence of hyperandrogenemia in the controls (Dunaif 2016).

A meta-analysis in mixed ancestries from multiple biobanks identified a novel locus associated with algorithmically defined Rotterdam PCOS cases from electronic health records (EHRs) (Zhang *et al.* 2020). Only one previously identified PCOS GWAS locus, ERBB4 (Day *et al.* 2015, 2018), was replicated, which is surprising given the replication of *FSHB* in multiple European ancestry PCOS cohorts (Day *et al.* 2015, 2018, Hayes *et al.* 2015). Recent studies have validated EHR-based algorithms for effectively identifying PCOS with positive predictive values over 90%, which should facilitate these biobank-based genetic analyses. Still, the best-performing algorithms have low sensitivity (Actkins *et al.* 2021). Therefore, EHR-predicted case cohorts would likely represent a distinct population of women with PCOS compared to traditionally defined PCOS case cohorts.

The largest GWAS have been meta-analyses conducted by the International PCOS Consortium. The first (Day *et al.* 2018) identified three novel loci along with 11 previously identified loci showing correlations with obesity, fasting insulin, T2D, lipid levels, and coronary artery disease, indicating shared genetic architecture between metabolic traits and PCOS. The *LHCGR, FSHR,* and *FSHB* genes are involved in gonadotropin production and secretion, which is perturbed in women with PCOS. The *INSR* gene is implicated in the pathogenesis of PCOS-related insulin resistance (Diamanti-Kandarakis & Dunaif 2012). Similarly, other loci such as *THADA* and *HMGA2* are T2D risk alleles. *DENND1A* is involved in androgen synthesis in theca cells, whereas *YAP1* plays a key role in folliculogenesis. *RAB5B*, *SUOX,* and *ERBB3* are risk alleles for type 1 diabetes. *RAD50* is involved in DNA damage repair and is associated with ANM.

**Figure 1:**
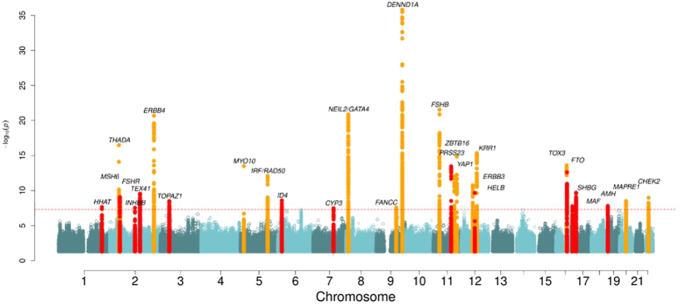
Manhattan plot showing the 29 genomic loci associated with PCOS. Variants within 300 kb on either side of a genome-wide significant signal are highlighted in red. The dotted line indicates the genome-wide significance level. Gene names indicate the consensus PCOS gene at each locus. (Reproduced with permission from Moolhuijsen *et al.* (2024)).

**Figure 2:**
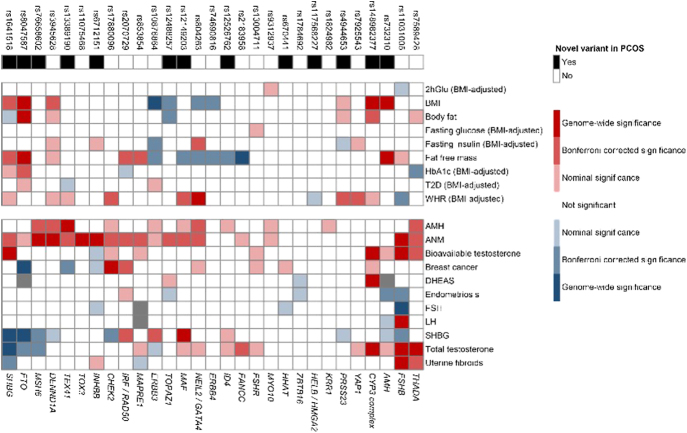
Heatmap of GWAS associations for the 29 PCOS loci with other relevant traits. Direction and strength of association between the 29 PCOS risk-increasing alleles with 20 other relevant traits and diseases with available GWAS summary statistics. Color coding indicates strength and direction (z-scores) of associations: positive (red) or negative (blue). In the upper row of the heatmap, novel PCOS loci identified in this study (black boxes) and previously reported loci (white boxes) are shown with corresponding lead variants. The upper panel and the lower panel of the heatmap show metabolic and reproductive phenotypes, respectively. Gray boxes indicate missing variant–trait association data. Genes are presented in the lower x-axis as ‘consensus gene’. BMI, body mass index; T2D, type 2 diabetes mellitus; WHR, waist-to-hip ratio; AMH, anti-Müllerian hormone; ANM, age at natural menopause; FSH, follicle-stimulating hormone; LH, luteinizing hormone; SHBG, sex hormone-binding globulin. (Reproduced with permission from Moolhuijsen *et al.* (2024)).

The most recent meta-analysis by the International PCOS Consortium expanded the number of genetic loci associated with PCOS from 16 to 29 and identified 31 associated plasma proteins (Fig. 1). Many risk-increasing loci were associated with later age at menopause, underscoring the reproductive longevity related to an increase in DNA repair and maintenance genetic variants in women with PCOS. The latter also might lead to increased oocyte numbers and/or availability across the lifespan and delayed reproductive aging. The proteomic analysis from that study highlighted perturbations of metabolically related biology that are typical in women with PCOS. A PCOS polygenic risk score was associated with adverse cardiometabolic outcomes, with differing contributions of testosterone and body mass index (BMI) in women and men. Moreover, this study concluded that although oligo- and anovulatory infertility are characteristic features of PCOS, no impact of PCOS susceptibility on childlessness was found (Fig. 2). The authors concluded that PCOS susceptibility confers balanced pleiotropic influences on fertility in women, and lifelong adverse metabolic consequences in both sexes (Moolhuijsen *et al.* 2024). Collectively, GWAS have substantially advanced our understanding of the pathophysiology of PCOS; however, additional fine mapping and functional studies are necessary to uncover specific mechanisms involved in PCOS (for extensive review see Dapas & Dunaif (2022)).

#### Mendelian randomization (MR)

MR assesses causal associations between PCOS risk and related exposure factors. MR analyses suggested variants associated with BMI, fasting insulin, menopause timing, depression, and male-pattern balding play a causal role in PCOS. This study also provided the first genetic evidence for a male phenotype for PCOS being premature baldness. Finally, this study also identified a causal link to depression and PCOS (Day *et al.* 2018). Indeed, MR provided evidence of causal associations of AMH serum concentrations, SHBG levels, menopausal age, adiposity, insulin resistance, depression, breast cancer, ovarian cancer, obsessive-compulsive disorder, and forced vital capacity with PCOS. Interestingly, no significant associations of type 2 diabetes mellitus, coronary heart disease, stroke, anxiety disorders, schizophrenia, bipolar disorders, and offspring birth weight with PCOS were found using MR (Wang *et al.* 2022, Zhu & Goodarzi 2022). The relationship between PCOS and delayed reproductive aging has also been studied in relation to the risk of breast cancer. Genome-wide cross-trait analysis identified a shared genetic basis between PCOS and estrogen receptor-positive breast cancer, probably due to prolonged estrogen exposure (Bi *et al.* 2024). Other studies revealed that specific fertility nutrients, such as omega-3 fatty acids, vitamin A, berberine, and curcumin, may reduce the risk of PCOS by improving metabolic and reproductive abnormalities associated with it (Shao *et al.* 2024, Zhang *et al.* 2024).

#### Next-generation sequencing

Although next-generation sequencing can identify rare variants, the low population frequencies of these variants require extremely large cohorts to detect their effects using standard case–control genetic association tests. Populations can be enriched for causal rare variants by studying individuals with extreme phenotypes or families with multiple affected members. Even in enriched cohorts, most rare alleles remain too infrequent to allow effective variant association testing (Dapas & Dunaif 2022).

Candidate gene next-generation sequencing of PCOS cases and reproductively normal control women identified 24 rare (MAF ≤1%) potentially deleterious coding variants in *AMH* (Gorsic *et al.* 2017). Of these variants, 18 were specific to cases. All but one of the PCOS-specific variants had significantly reduced AMH signaling, whereas none of the genetic variants observed in control subjects showed defects in signaling (Gorsic *et al.* 2017). In a second study (Gorsic *et al.* 2019), rare noncoding variants in *AMHR2* as well as *AMH* were identified that also decreased signaling. Additional studies found that the *AMH* P151S and H506Q variants disrupted normal processing and secretion of AMH, causing endoplasmic reticulum retention. These *AMH* variants should be considered when serum AMH levels are relatively low in PCOS cases (Meng *et al.* 2023). A SNP in the promoter region of the *AMH* has also been linked to lower AMH levels both in healthy controls and in women with PCOS (Moolhuijsen *et al.* 2022).

Unlike candidate gene sequencing, which relies on prior biological hypotheses to select genes of interest, whole-genome sequencing (WGS) takes an unbiased, hypothesis-free approach, allowing for genome-wide association testing to identify previously unrecognized genetic contributors to disease. By examining the entire genome, WGS enables the discovery of both common and rare variants associated with PCOS, independent of pre-existing assumptions about disease mechanisms (Dapas & Dunaif 2020).

A family-based WGS of 261 individuals from 62 families with one or more daughters diagnosed with PCOS applied this agnostic approach to identify gene–level associations of rare variants (MAF ≤2%) with the disorder (Dapas *et al.* 2019). To increase statistical power in detecting PCOS-associated variants, rare variants were also tested against quantitative traits related to PCOS, including testosterone (T), dehydroepiandrosterone sulfate (DHEAS), insulin, glucose, LH, FSH, and SHBG, integrated into a meta-statistic, allowing for the combination of multiple association tests while minimizing the penalty for multiple hypothesis testing. This approach identified a set of rare variants in the *DENND1A* gene that were significantly associated with alterations in reproductive and metabolic traits within affected families.

*DENND1A* was initially implicated in PCOS through GWAS (Shi *et al.* 2012) and was later shown to regulate androgen production in ovarian theca cells through its splicing isoform, *DENND1A.V2* (McAllister *et al.* 2014). Notably, the rare variants identified in the family-based study were not in linkage disequilibrium with previously reported *DENND1A* GWAS variants (Chen *et al.* 2011, Shi *et al.* 2012, Day *et al.* 2018), which have no apparent functional impact on splicing or transcription (McAllister *et al.* 2014, Tee *et al.* 2016). However, the rare variants were predicted to disrupt transcription factor binding and RNA-binding protein motifs, suggesting a potential functional role. Despite this, targeted sequencing (Eriksen *et al.* 2013, McAllister *et al.* 2014, Tee *et al.* 2016) and whole-exome sequencing (WES) (Khan *et al.* 2018) have not yet identified *DENND1A* variants associated with PCOS or *DENND1A.V2* expression. However, these studies were limited to specific gene regions and small case–control cohorts. Further replication and functional analyses are required to determine whether the rare *DENND1A* variants observed in the family study influence *DENND1A.V2* expression or contribute to PCOS susceptibility. Notably, one or more rare *DENND1A* variants were present in individuals from 50% of the families analyzed (Dapas *et al.* 2019). These findings support a genetic model in which individual causal variants are rare, but together they occur frequently in genes associated with the disease.

While no other gene associations reached genome-wide significance in the family-based meta-analysis, two of the top five associated genes are established PCOS candidate genes. *C9orf3*, a gene previously identified in PCOS GWAS (Chen *et al.* 2011, Shi *et al.* 2012, Hayes *et al.* 2015, Day *et al.* 2018), and *BMP6*, which plays a role in folliculogenesis by regulating granulosa cell function (Shi *et al.* 2011) and is overexpressed in individuals with PCOS (Khalaf *et al.* 2013), were among the strongest associations. These genomic sequencing studies collectively reinforce these genes’ role in PCOS pathogenesis and suggest that rare, family-specific variants affecting key genes may contribute substantially to PCOS risk.

Other studies of rare genetic variants in PCOS remain limited due to small sample sizes, methodological constraints, and a lack of functional validation. WES in limited cohorts has identified candidate variants in *FBN3*, *FN1* (fibronectin 1), *FSHR* (follicle-stimulating hormone receptor), *SCARB1* (scavenger receptor class B member 1), and *INSR*, but their pathogenicity remains uncertain (Karakaya *et al.* 2022, Janani *et al.* 2023). *FBN3*, initially linked to PCOS through family-based studies (Stewart *et al.* 2006, Urbanek *et al.* 2007), is expressed in the ovarian extracellular matrix (Prodoehl *et al.* 2009). Still, as a large gene, it is prone to sequencing artifacts and false-positive variant calls (Fuentes Fajardo *et al.* 2012). Similarly, rare exonic variants in metabolic genes, including *UCP1*, *UCP2*, *IRS1*, and *GHRL*, have been associated with higher BMI and fasting insulin levels in PCOS, though replication is needed (Sharma *et al.* 2023*b*). A study sequencing *LMNA* (Lamin A/C) found eight missense variants in 15 out of 811 PCOS cases, which were associated with extreme insulin resistance and high triglyceride levels, with replication in an independent cohort (Bauer *et al.* 2024).

A key limitation of WES is its restriction to coding regions, excluding noncoding regulatory variants that likely contribute to PCOS. Most GWAS-identified risk variants map to noncoding regions, underscoring the importance of regulatory elements in disease susceptibility (Dapas & Dunaif 2022). In addition, reliance on *in silico* prediction tools for variant classification is problematic, as computational models often misclassify variants as deleterious without experimental validation. Studies of *AMH* gene variants in PCOS (Gorsic *et al.*) illustrate this issue, where only a subset of predicted deleterious variants exhibited functional consequences. Without biochemical or cellular assays, the true impact of rare variants remains uncertain. These limitations highlight the need for functional analyses to establish the role of rare variants in PCOS pathogenesis (Dapas & Dunaif 2020).

### Epigenetic and environmental influences

In addition to genetics, epigenetic mechanisms have been proposed to contribute to the etiology of PCOS (Stener-Victorin & Deng 2021). Epigenetics refers to modifications of the DNA affecting mechanisms that control transcriptional regulation, such as DNA methylation, histone modifications, and non-coding RNAs (Lee *et al.* 2014). According to the Developmental Origins of Health and Disease hypothesis, altered epigenetic programming is associated with adult onset of several metabolic diseases and can originate from an adverse uterine environment (Aiken & Ozanne 2014). Therefore, epigenetics has been suggested as a missing link in PCOS, connecting genetics and the environment, such as *in utero* exposure to altered hormone levels, nutritional factors, and environmental factors. For example, the offspring of women with PCOS are exposed to increased androgen and AMH levels *in utero* (Peigne *et al.* 2023), and both female and male offspring have an increased risk of metabolic diseases (Risal *et al.* 2019, 2023, Chen *et al.* 2021, Mimouni *et al.* 2021). Supported by animal models of PCOS, epigenetics is also suggested to contribute to a potential transgenerational inheritance pattern of PCOS (Xu *et al.* 2014, Nilsson *et al.* 2018, Risal *et al.* 2019, Mimouni *et al.* 2021).

Most studies addressing epigenetic modifications in PCOS have addressed the DNA methylation profile. Changes were observed in various tissues, including granulosa cells and adipose tissue (Sagvekar *et al.* 2019, Zhao *et al.* 2021, Divoux *et al.* 2022). According to a systematic review and meta-analysis, significant global hypomethylation of DNA is observed in women with PCOS. An analysis of 20 specific genes associated with PCOS, including *AMH, AMHR2, YAP1, CYP19A1*, and *LHCGR*, showed that some genes are hypomethylated, whereas others are hypermethylated (Rawat *et al.* 2022). In a study by Wang *et al.* (2023), several differentially methylated regions were identified in blood samples of women with PCOS. Pathway enrichment analysis indicated an overlap with *THADA*, *AOPEP*, *TRIML2*, *KCNA4*, and *FDFT1*. Combined, these studies suggest that changes in methylation status may affect known PCOS susceptibility genes.

Histone modification, involving acetylation, methylation, phosphorylation, ubiquitylation, or SUMOylation, has not been addressed extensively in PCOS. However, analysis of cumulus cells of women with PCOS revealed an altered pattern of histone markers of the *CYP19A1* promoter (Hosseini *et al.* 2019). Furthermore, a recent study showed that expression of the deacetylase *HDAC1* is reduced in serum samples of women with PCOS (Chen *et al.* 2024). More insight into the potential involvement of histone modification in PCOS stems from animal models of PCOS. In agreement with findings in women with PCOS, prenatal DHEA exposure of female mice resulted in the downregulation of *HDAC1*. Interestingly, the overexpression of this deacetylase alleviated the DHEA-induced ovarian PCOS-like phenotype (Chen *et al.* 2023). In prenatally testosterone-exposed sheep, the expression of epigenetic enzymes affecting both gene-activating (H3K4me3) and repressive (H3K27me3) histone modifications was altered (Sinha *et al.* 2020). Furthermore, in granulosa cells, the expression of the histone methyltransferases SUV39H1 and EZH2 was increased, while the deacetylase HDAC3 and demethylase KDM1A expression decreased. These androgen-induced modifications were cell-specific and depended on the timing of androgen exposure (Guo *et al.* 2019), limiting direct translation to women with PCOS.

Non-coding RNAs (ncRNAs), including microRNAs (miRNAs) and long non-coding RNAs (lncRNAs), play a role in the regulation of gene transcription and post-transcriptional translation (Kaikkonen *et al.* 2011). Based on a systematic review and meta-analysis, miRNA profiles in serum, follicular fluid, granulosa cells, theca cells, and adipose tissue are altered in women with PCOS (Mu *et al.* 2021). For instance, increased expression levels of miR-30c, miR-146a, and miR-222 levels were observed in serum of women with PCOS, and these miRs are suggested as potential diagnostic markers (Long *et al.* 2014, Hosseini *et al.* 2017). In adipose tissue, increased expression of miR-93 has been reported (Chen *et al.* 2013, Wu *et al.* 2014). However, overall the consistency between studies is small (Mu *et al.* 2021). While initial studies used a targeted approach analyzing specific miRNAs due to their involvement in follicle growth and insulin resistance, recent studies have taken an unbiased approach by applying whole transcriptome sequencing to assess the total ncRNA profile. Comparing granulosa cells of women with PCOS with controls, 3,000 differentially expressed lncRNAs were identified, which, based on pathway analysis, have a regulatory role in mitochondrial function (Jin *et al.* 2018). LncRNA NONHSAT102254, associated with the gene corticotropin-releasing factor-binding protein (*CRFBP*), was the strongest upregulated lncRNA in this study. Using existing Gene Expression Omnibus databases, gene-miRNA interaction networks were identified with *VEGFA* (miR-126-3p), *SOX2* (miR-126-3p), *KRAS* (miR-183-5p), *AKT1* (miR-126-3p), and *SMAD4* (miR-183-5p; miR-933) as hub genes and the involvement of an altered immune regulation in women with PCOS (Bhandary *et al.* 2024, Quan *et al.* 2024). In addition, within different PCOS phenotypes, differences in specific miRNA expression (miR-23a-3p, miR-424-5p) were observed (Trummer *et al.* 2024), suggesting that ncRNAs may contribute to different PCOS phenotypes.

It should be noted that most findings related to epigenetics in women with PCOS are based on a small sample size, without considering the different PCOS phenotypes. In addition, the presence or absence of a metabolic phenotype has not been fully addressed. Therefore, further studies are needed to determine the role of different epigenetic mechanisms in PCOS. Integrating multi-omics approaches to explore gene–environment interactions may be the next step in deciphering the etiology of PCOS.

### Resolving the heterogeneity of PCOS – data-driven subtyping

Many chronic diseases are complex traits arising from interactions between susceptibility genes and environmental factors. These conditions are often defined by arbitrary diagnostic criteria and may represent the phenotypic convergence of multiple genetic etiologies under a single clinical diagnosis (Flint & Kendler 2014, Ringman *et al.* 2014, von Coelln & Shulman 2016, Ahlqvist *et al.* 2018, Udler *et al.* 2018, Misra *et al.* 2023). The heterogeneity of PCOS has long been recognized, yet traditional classification systems based on expert-defined diagnostic criteria fail to delineate biologically distinct subtypes. Genetic studies demonstrate that PCOS cases defined by different diagnostic criteria share a largely similar genetic architecture, indicating that these classifications do not capture meaningful etiological differences (Dapas & Dunaif 2022).

**Figure 3:**
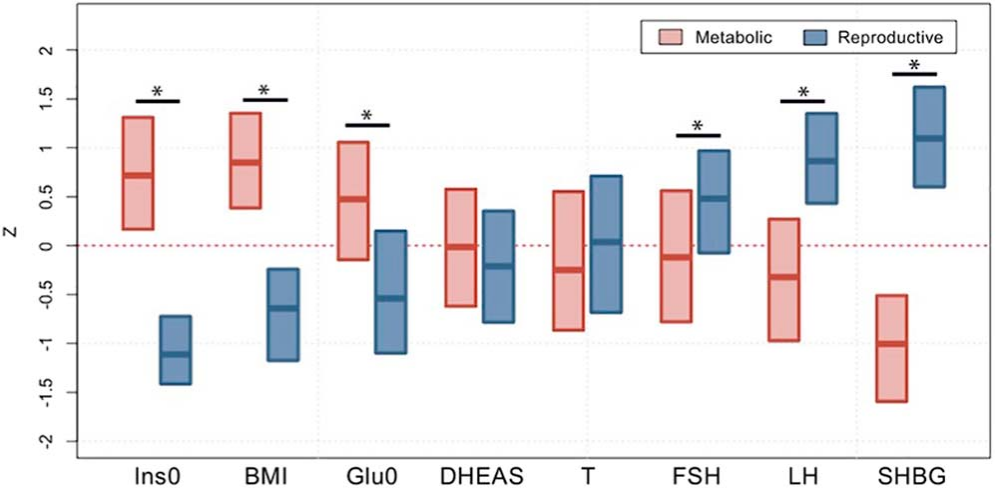
Phenotypic trait distributions in reproductive and metabolic subtypes. Median and IQRs are shown for normalized, adjusted quantitative trait distributions of genotyped PCOS cases with reproductive or metabolic subtype. The figure illustrates the traits for which the subtypes differ significantly with asterisk (*Bonferroni-adjusted Wilcoxon, *P*_adj_ < 0.05): Ins0, BMI, Glu0, FSH, LH, and SHBG. BMI, body mass index; DHEAS, dehydroepiandrosterone sulfate; FSH, follicle-stimulating hormone; Glu0, fasting glucose; Ins0, fasting insulin; IQR, interquartile range; LH, luteinizing hormone; PCOS, polycystic ovary syndrome; SHBG, sex hormone-binding globulin; T, testosterone. (Reproduced with permission from Dapas *et al.* (2019)).

Machine learning approaches, mainly unsupervised clustering methods, offer a promising strategy for resolving this heterogeneity by identifying patterns within high-dimensional phenotypic data. Using hierarchical clustering analysis of PCOS quantitative traits, distinct, reproducible subtypes have been identified and designated as: i) reproductive, characterized by elevated LH and SHBG; ii) metabolic, defined by higher BMI, fasting insulin, and fasting glucose; and iii) background, representing cases without a clear phenotypic pattern (Dapas *et al.* 2020). Subtype-specific GWAS revealed that each subtype is associated with predominantly novel, genome-wide significant loci, supporting distinct genetic architectures (Dapas *et al.* 2020). These subtype-specific genetic associations provide independent validation that clustering captures biologically meaningful variation in PCOS (Tahir & Gerszten 2020). These findings suggest that PCOS is not a single disorder but a syndrome encompassing multiple genetic and phenotypic subtypes with divergent pathogenic mechanisms (Fig. 3).

The robustness of this classification has been demonstrated through replication in independent cohorts of European ancestry from the Netherlands and Greece, as well as in an East Asian South Korean ancestry cohort (Brewer *et al.* 2022). Some subtype-associated genetic loci are present trans-ethnically, further supporting the validity and generalizability of this classification (Brewer *et al.* 2023). Unlike subtyping efforts in T2D, which have faced challenges with replication and generalizability (Stefan & Schulze 2023, Varghese *et al.* 2023), PCOS subtypes have proven stable across populations and provide a foundation for gene discovery using hard-clustering approaches.

Beyond genetic differentiation, PCOS subtypes display distinct phenotypic characteristics that extend beyond the traits used for clustering. The reproductive subtype is further associated with higher AMH levels and increased follicle counts, while the metabolic subtype exhibits higher low-density lipoprotein levels and elevated blood pressure (van der Ham *et al.* 2024). A study using the published PCOS phenotypic clustering algorithm (Dapas *et al.* 2020) found that the reproductive subtype was associated with an increased risk of ovarian hyperstimulation during assisted reproduction. In contrast, the metabolic subtype was linked to poorer embryo quality and lower implantation success (Chen *et al.* 2024).

Furthermore, independent clustering approaches using alternative methodologies have identified comparable subtypes. An analysis integrating GWAS summary statistics and clinical traits delineated four PCOS genetic clusters with distinct top loci, including an obesity-driven (*FTO*), reproductive (*FSHB*), inflammatory (*ATXN2* and *SH2B3*), and metabolic (*MAF* and *SLC38A11*) cluster. Notably, these genetic clusters were also associated with differential clinical outcomes, such as an increased risk of type 2 diabetes in the obesity-driven cluster (Stamou *et al.* 2024).

Subtyping through unsupervised clustering has become an important approach to resolving heterogeneity in other complex traits, most notably T2D, where distinct genetic and metabolic subtypes have been identified with implications for disease progression and treatment response (Udler *et al.* 2018, Mansour Aly *et al.* 2021). This emerging classification framework represents a paradigm shift in PCOS research, moving beyond subjective, criteria-based diagnosis toward an objective, biologically informed approach. By identifying subtypes with unique genetic architectures and metabolic profiles, data-driven classification provides critical insights into PCOS pathophysiology, refines disease classification, guides targeted therapeutic strategies, and lays the foundation for precision medicine in PCOS.

### Genetic architecture of PCOS

Genetic studies have established a strong hereditary component in PCOS pathogenesis, with GWAS identifying numerous common risk alleles associated with neuroendocrine, reproductive, and metabolic pathways. However, GWAS SNPs primarily tag genomic regions rather than representing causal variants themselves (Visscher *et al.* 2017). Fine-mapping studies combined with functional analyses are required to determine how genetic variation within these loci contributes to PCOS. Epigenetic mechanisms, including differential DNA methylation and noncoding RNA regulation, may also interact with genetic variation to influence PCOS phenotypes (Risal *et al.* 2019, Mimouni *et al.* 2021).

Genetic risk loci identified in association studies are often population-specific due to ancestral differences in allele frequencies and linkage disequilibrium patterns. Although PCOS GWAS in European and Han Chinese cohorts have revealed shared genetic risk loci, the specific SNPs associated with PCOS differ between populations (Mutharasan *et al.* 2013, Day *et al.* 2018, Laven 2019). Rare genetic variation appears to be even more population-specific, necessitating multiethnic studies for fine-mapping causal variants (Qin *et al.* 2020). Population differences in linkage disequilibrium structure can further aid in pinpointing causal variants within shared risk loci (Asimit & Zeggini 2011).

PCOS genetics align with the omnigenic model of complex traits, where heritability is driven by an extensive polygenic network of peripheral genes with small, indirect effects on phenotype (Boyle *et al.* 2017, Liu *et al.* 2019, Mathieson 2021). Core genes, by contrast, have direct functional roles and appear enriched for rare variants associated with disease. Supporting this model, rare coding and noncoding variants in *AMH*, *AMHR2*, and *DENND1A* have been identified in PCOS cases, implicating AMH signaling and androgen biosynthesis as core reproductive pathways in PCOS pathogenesis.

A fundamental advance in PCOS research has been using unsupervised machine learning approaches to resolve disease heterogeneity. Traditional diagnostic criteria fail to delineate biologically distinct subtypes, as GWAS demonstrate that PCOS cases defined by NIH, Rotterdam, and self-reported criteria share a similar genetic architecture (Day *et al.* 2018). In contrast, machine learning-driven classification based on hormonal profiles has identified reproductive and metabolic subtypes with unique genetic architectures (Dapas *et al.* 2020). These subtypes are associated with distinct risk loci and core biological pathways, providing an objective framework for understanding PCOS pathogenesis. Furthermore, rare genetic variants in *DENND1A*, *AMH*, and *AMHR2* support androgen biosynthesis and AMH signaling as core causal pathways. Environmental risk factors likely interact with these genetic susceptibilities, with specific phenotypes arising from distinct prenatal exposures (Roland *et al.* 2010, Houten *et al.* 2014, Tata *et al.* 2018).

This data-driven subtyping approach represents a paradigm shift in PCOS classification, moving from expert opinion-based diagnosis to an objective system rooted in biological differences. By integrating genetic, epigenetic, and environmental factors, this model provides a foundation for precision medicine, facilitating the development of targeted interventions tailored to distinct PCOS subtypes.

## Conclusions and future perspectives

Distinct subtyping will lead to a paradigm shift in PCOS classification, moving from expert opinion-based diagnosis to an objective system rooted in biological differences. By integrating genetic, epigenetic, and environmental factors, this model provides a foundation for precision medicine, facilitating the development of targeted interventions tailored to distinct PCOS subtypes.

## Declaration of interest

YVL: received an internal research grant from the Erasmus MC (The Synergy grant), and she received fees from Ferring and Merck for presentations. JAV: received royalties from AMH assays, paid to the institute/laboratory with no personal financial gain. AD: is a consultant for Quest Diagnostics, Inc, and AcaciaBio, Inc. JSEL: reports grants from Ansh Labs, Ferring, Roche Diagnostics, Merck, and personal fees from Ferring, Titus Healthcare, Gedeon Richter, Ansh Labs, and Roche Diagnostics. He is an unpaid board member and the past president of the AE-PCOS Society.

## Funding

This research did not receive any specific grant from any funding agency in the public, commercial, or not-for-profit sector.

## Author contribution statement

YVL, JAV, and AD were responsible for writing, editing, reviewing, and approving the final version. JSEL contributed to conceptualization, writing, editing, reviewing, and approving the final version.
